# The lived experiences of rural women diagnosed with the human immunodeficiency virus in the antenatal period

**DOI:** 10.1080/17290376.2017.1379430

**Published:** 2017-09-26

**Authors:** Genevieve Marion Fords, Talitha Crowley, Anita S van der Merwe

**Affiliations:** ^a^ Department of Nursing and Midwifery, Faculty of Medicine and Health Sciences, Stellenbosch University, Francie van Zijl Drive, Cape Town 8000, South Africa

**Keywords:** HIV, pregnancy, lived experiences, rural, VIH, grossesse, expériences vécues, rural

## Abstract

*Background:* In South Africa, pregnant women are diagnosed with human immunodeficiency virus (HIV) at antenatal clinics and simultaneously initiated on antiretroviral treatment (ART). An HIV diagnosis together with the initiation of ART has an emotional impact that may influence how pregnant women cope with pregnancy and their adherence to a treatment plan. The aim of the study was to explore the lived experiences of women diagnosed with HIV in the antenatal period in a rural area in the Eastern Cape province of South Africa. *Methods:* A qualitative approach with a descriptive phenomenological design was utilised. The study applied purposive sampling to select participants from a local community clinic in the Eastern Cape. Ten semistructured interviews were conducted, transcribed and analysed using Colaizzi's framework. *Results:* Four themes formed the essential structure of the phenomenon being investigated: a reality that hits raw, a loneliness that hurts, hope for a fractured tomorrow and support of a few. Although the participants had to accept the harsh reality of being diagnosed with HIV and experienced loneliness and the support of only a few people, they had hope to live and see the future of their children. *Conclusion:* Women diagnosed with HIV during pregnancy are ultimately concerned with the well-being of their unborn children, and this concern motivates their adherence to ART. Women's lived experiences are situated in their unique sociocultural context, and although some known challenges remain, counselling and support strategies need to be informed by exploring context-specific issues and involving the local community.

## Résumé

*Contexte:* En Afrique du Sud, les femmes enceintes sont diagnostiquées avec le virus de l'immunodéficience humaine (VIH) dans les cliniques prénatales et simultanément initiées au traitement antirétroviral (ARV). Le diagnostic du VIH suivi de l'initiation au traitement ARV a un impact émotionnel qui peut influencer la façon dont les femmes enceintes font face à la grossesse et leur adhésion au plan de traitement. Le but de cette étude était d'explorer les expériences vécues des femmes diagnostiquées avec le VIH dans la période prénatale dans une région rurale de la province de Eastern Cape, en Afrique du Sud. *Méthodes:* Une approche qualitative avec une conception phénoménologique descriptive a été utilisée. L'étude a appliqué un échantillonnage intentionnel pour sélectionner les participants d'une clinique communautaire locale dans la Province de Eastern Cape. Dix interviews semi-structurées ont été menées, transcrites et analysées à l'aide du cadre conceptuel de Colaizzi. *Résultats:* Quatre thèmes ont constitué la structure essentielle du phénomène étudié: une réalité qui frappe, une solitude qui fait mal, l'espoir d'un demain fracturé et le soutien de quelques-uns. Bien que les participants aient dû accepter la dure réalité d'être diagnostiqués avec le VIH et avoir connu la solitude et le soutien de peu de personnes, ils avaient l'espoir de vivre et de voir l'avenir de leurs enfants. *Conclusion:* Les femmes diagnostiquées avec le VIH pendant la grossesse sont finalement concernées par le bien-être de leurs enfants à naître, et cette préoccupation motive leur adhésion au traitement ARV. Les expériences vécues par les femmes se situent dans leur contexte socioculturel unique et, pendant que certains défis connus persistent, les stratégies de conseil et de soutien doivent être informées en explorant les problèmes spécifiques au contexte et en impliquant la communauté locale.

## Introduction and background

An increasing number of pregnant women in low- and middle-income countries are being tested for human immunodeficiency virus (HIV) during pregnancy. In 2014, 62% of pregnant women living with HIV received antiretroviral treatment (ART) (UNICEF, 2016). Of the 1.5 million pregnant women estimated to live with HIV, 90% reside in sub-Saharan Africa. South Africa has a high burden of HIV, with a national antenatal HIV prevalence of 29.7% (National Department of Health, 2013).

In South Africa, prevention of mother-to-child transmission (PMTCT) guidelines have evolved over time to incorporate emerging evidence. Guidelines recommend HIV testing in the antenatal period and, more recently, initiating lifelong triple ART as soon as the diagnosis has been made. This PMTCT regime is known as Option B+. The rationale for expedited lifelong treatment initiation in pregnancy is to optimise the reduction in HIV transmission that ART affords the baby while also improving maternal health outcomes (World Health Organization, 2013).

According to the South African National Antenatal Sentinel HIV Prevalence Survey (National Department of Health, 2013), there was a higher HIV prevalence, above the national average of 29.7% (95% CI: 28.9–30.2), amongst women older than 25 who were aware of their status before participating in the survey. This may be because they were tested before in pregnancy. However, many women are not aware of their HIV status when tested in pregnancy. An HIV diagnosis together with the initiation of ART may have an emotional effect on pregnant women that influences how they will cope with the pregnancy as well as their adherence to a treatment plan (Black et al., 2014; Kasenga, Hurtig, & Emmelin, 2010). A meta-analysis by Nachega et al. (2012) found suboptimal antepartum and postpartum ART adherence among pregnant women in low-, middle- and high-income countries. Loss to initiation of ART, missed visits, disengagement from care and treatment interruptions are common during antenatal and postnatal care for HIV-positive pregnant women in South Africa (Phillips et al., 2014; Schnippel, Mongwenyana, Long, & Larson, 2015). This may impair the effectiveness of PMTCT programmes and worsen maternal and infant health outcomes.

Recent studies have reported on barriers to and facilitators of initiation and adherence within PMTCT programmes in both rural and urban settings. Qualitative studies in Malawi found that although women had positive perceptions of Option B+ during pregnancy, initiating ART on the same day as HIV diagnosis was problematic since many wanted to discuss the diagnosis with their husbands, felt healthy or needed time to think (Katirayi et al., 2016; Kim et al., 2016). Other barriers to participation in PMTCT programmes include fears of HIV disclosure to partners or husbands, lack of partner support, community-based HIV-related stigma, poor relationships with healthcare workers and HIV stigmatising practices in healthcare settings (Elwell, 2016; Greene, Ion, Kwaramba, Smith, & Loutfy, 2016).

Growing confidence in the effectiveness of ART, the better side effect profile of current ART regimens and the positive benefits of ART, such as having an HIV uninfected baby and prolonging her life to fulfil mothering responsibilities, are reported facilitators of ART for pregnant women (Black et al., 2014; Elwell, 2016; Katirayi et al., 2016; Treffry-Goatley et al., 2016).

A systematic review by Gourlay, Birdthistle, Mburu, Iorpenda, and Wringe (2013) of the barriers to and facilitators of the uptake of ART for PMTCT found that several known health system barriers and community-level factors continued to hinder optimal participation in PMTCT programmes. This necessitates community-driven and patient-centred approaches that are sensitive to the local context.

More studies on the factors affecting ART uptake for PMTCT have been conducted in urban compared to rural settings. Key issues that affect pregnant women in rural areas include distances to clinics, frequency of clinic visits and culture-specific influences (Gourlay et al., 2013). Treffry-Goatley et al. (2016) allude to the complexity of the issues that individuals and families affected by HIV in a rural South African community face. These may require novel approaches to support optimal adherence to ART. Only a few studies in South Africa have focused on the experiences of pregnant women in rural settings who have tested HIV positive when they became pregnant (Nkonki et al., 2007; Peltzer, Mosala, Dana, & Fomundam, 2008). These studies were conducted before the implementation of Option B+.

Recommendations for improving PMTCT uptake in rural areas include peer education, counselling support, increased male partner involvement, the use of community care workers or lay counsellors for follow-up and screening for gender-based violence (Jones et al., 2015). In rural Zimbabwe, the use of mother support groups did not significantly improve retention in care rates amongst HIV-exposed infants (Foster et al., 2017), while in rural South Africa, male involvement alone without an active couples psychoeducational intervention did not improve PMTCT outcomes (Weiss et al., 2014). It is therefore evident that what works in one setting may not necessarily be translated to other settings and single interventions may not be as effective as multiple interventions.

A better understanding of the lived experiences of women diagnosed with HIV during pregnancy in rural contexts such as some parts of the Eastern Cape and how such a diagnosis influences their behaviour may further elucidate which interventions may be most appropriate to improve PMTCT uptake in this setting.

## Aim

The aim of the study was to explore the lived experiences of women who had been diagnosed with HIV for the first time during the antenatal period and who resided in the rural Eastern Cape.

## Methods

### Research design

The researcher followed a qualitative descriptive phenomenological study approach, which according to Watson, Mckenna, Cowman, and Keady (2008) is an approach that describes the lived experiences of the participants and explores the possible hidden meaning behind the experiences described by the participants. This approach was chosen as a way to gain access to the inner world of women to understand their thoughts and feelings about being diagnosed with HIV during pregnancy and how this influenced their relationships and decisions about participation in the PMTCT programme. To ensure that the genuine lived experiences were captured by the researcher, the study was based on the Husserlian philosophy of phenomenology, using bracketing. This process of bracketing is a continuous process to ensure that the true phenomenon under investigation is not influenced by the researcher (Grove, Burns, & Gray, 2013). The researcher kept a reflexive journal of preconceived ideas and assumptions that were based on her experience working as a professional nurse in a conscious effort to limit the influence it may have on the data collection and analysis processes. Bracketing was also done by not doing an in-depth review of the literature before data collection and analysis.

### Setting

According to Statistics South Africa (2013), the Eastern Cape province has the highest fertility rate in South Africa (2.7%). The province also experiences the highest amount of provincial migration (Statistics South Africa, 2013) and has an HIV antenatal sero-prevalence of 31.4%, which is above the national average (National Department of Health, 2013). The study focused on pregnant women residing in the Maluti local service area in the Eastern Cape who attended one of the local clinics. In this setting, antenatal HIV testing and counselling is done by lay counsellors after which women return to the professional nurses for further antenatal care.

### Population and sampling

One hundred and nine women were diagnosed with HIV in the antenatal period from January 2015 to July 2015 and formed the study population. The researcher purposively sampled 10 women who met the study inclusion criteria. These women had to be over the age of 18, must have been diagnosed with HIV for the first time in the antenatal period of pregnancy, must have been on ART for at least two months and had to be attending the local community clinic for their antenatal or postnatal care. The sample size in the study depended on when data saturation had been established. Data saturation was reached at the ninth interview when no new categories or themes emerged.

### Data collection

The professional nurse who provided antenatal care at the clinic identified and approached the HIV-positive pregnant women and requested their permission for referral to the researcher in order to keep their HIV status confidential. The researcher evaluated whether referred participants met the study inclusion criteria, informed them about the study and obtained written consent. Data collection occurred through individual interviews by the first author, using a semistructured interview guide with open-ended questions.

A pilot interview was conducted with one participant who met the inclusion criteria. Since the interview guide questions did not change and to ensure that the participant's voice was heard, data from this interview were included in the analysis. Following the pilot study, another nine interviews were conducted in a private room in the clinic, following informed consent from participants. All participants were interviewed in English at the preference of the participants, although an interpreter was available for participants who preferred to use a language other than English, to ensure the information given by the participant was accurately captured and understood. However an interpreter was not used in the study as the participants all preferred English and were able to express themselves adequately. They were encouraged to provide full descriptions of their experiences, which included their feelings, images, sensations, memories as well as the situation in which their experiences occurred. Interviews were between 30 and 60 minutes in duration, audio-recorded and transcribed verbatim. Following data analysis, the researcher individually discussed the findings with 6 of the 10 participants. The themes resonated with the participants, and they did not have any new information to add. Four of the participants were not available for validation.

The trustworthiness of the research study was ensured by the application of the four principles of trustworthiness described by Lincoln and Guba in Brink, Van der Walt, and Van Rensburg (2012): (i) credibility, (ii) dependability, (iii) transferability and (iv) confirmability. This was done through member checking of the findings with participants and thick descriptions substantiated with verbatim quotations. The researcher had regular debriefing sessions with the study supervisors who checked the coding, themes and essential structure of the phenomenon under investigation. An audit trail was kept should independent review be required.

### Data analysis

Colaizzi's seven-step method, described by Mackenzie (2009), was used to guide the data analysis process. This method is one of the most often-used methods of data analysis in descriptive phenomenological enquiry and is in essence concerned with describing everyday human existence. The first author transcribed the interviews personally to obtain a sense of the whole. Significant statements were extracted from each transcript, and meanings were formulated. Meanings were organised into clusters of themes, and these were validated by referring to the original transcripts. A comprehensive description of the phenomenon being investigated was written down, and the essential structure was formulated. Finally, the first author returned to the participants to confirm that the analysis described their true experiences.

### Ethical considerations

Approval for the study was granted by the Health Research Ethics Committee of Stellenbosch University (Ethics reference: S14/10/245). Permission for conducting the study was also obtained from the Eastern Cape Department of Health as well as the manager of the local community clinic. All participants in the study were individually provided with information about the research project by the researcher and gave written consent for participation in the study. The human rights of participants were protected by ensuring their right to self-determination; confidentiality and anonymity; protection from discomfort and harm; and fair treatment and justice. Although the study topic was sensitive, participants expressed relief and had a positive experience of sharing their stories. The first author shared the findings with the participants who were available at the time and with healthcare workers and managers at the clinic and sub-district.

## Results

### Participant characteristics

The study sample consisted of 10 participants. One of these participants was interviewed in the pilot study. All participants were clients attending the local community clinic and had been diagnosed with HIV for the first time during the antenatal period of pregnancy. Six participants were postnatal clients and four were antenatal clients. Participants were from various rural communities except two participants who lived in the local town. Eight participants were not married and two were married. Of the eight single participants, three were in a stable relationship with their partners while five were not in a relationship. All participants had children prior to the present pregnancy and had tested HIV negative in previous pregnancies. Participants were between the ages of 24 and 46.

### Essential structure of the phenomenon being investigated

**Fig. 1.**
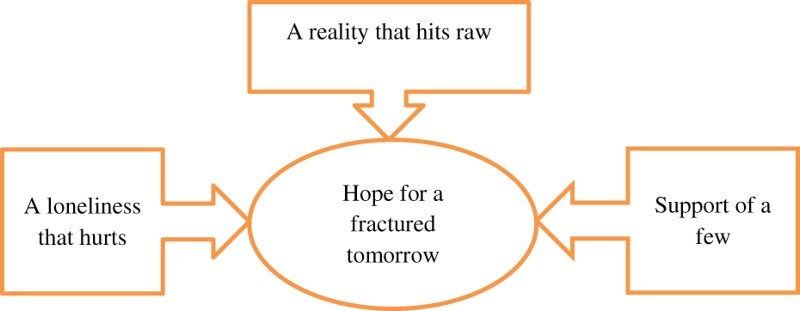
Essential structure of the phenomenon.

Four key themes and several subthemes emerged that formed the essential structure of the phenomenon (Fig. 1).

Although the participants had to accept the harsh reality of being diagnosed with HIV and experienced loneliness and the support of only a few people, they had hope to live and see the future of their children.

#### A reality that hits raw

The reality of an HIV diagnosis and the consequences that it had for their future and their current pregnancy were difficult to accept. Participants expressed disillusionment since they had felt that they would never become HIV positive. Some thought that they were safe from HIV since they only had one partner and that they would be able to avoid HIV infection.

I thought this could never happen to me. That's what I honestly thought. It will never happen to me. I’m too wise for this to happen to me. (P8)

I was shocked because I didn't know where I got HIV from. (P2)

I was angry because I knew myself. The first child was negative and I was negative. So something must have happened in between. (P1)

They described going home after the news as an unbearable time and did not want to be alone as it preoccupied their thoughts. They went to friends in an attempt to avoid thinking about what had happened at the clinic. Yet, they relived the diagnosis in their mind and it haunted them.

I can't sleep, it comes to me, I’m HIV positive. How is life going to be, you know? (P7)

Even after receiving the diagnosis, some questioned whether they were really HIV positive.

Even when she was giving me my results … is this my CD4 count, really? She said ‘Yes it is’. But I was so healthy, healthy and healthy and I couldn't believe how. Is this true? (P5)

Receiving the news changed the feelings that some participants had towards their pregnancy and the unborn child that they were carrying. Consequently, they considered abortion as an option as they thought that terminating the pregnancy would improve their situation. Some felt that an HIV diagnosis changed how they saw themselves and described themselves as being different. This manifested in a negative self-image.

The reality of the HIV diagnosis caused some participants to value the time spent with loved ones as they felt that they had limited time to live.

I can't even be away from these children. I’m thinking if I die tomorrow. I can't miss a second with them. I’ve never been apart from Adam [pseudonym]. I’ve never, not once gone out. For the same reason. I don't want to lose out on his life. (P8)

#### A loneliness that hurts

Participants described their lives after an HIV diagnosis as filled with loneliness – a loneliness that hurt as it was for them not possible that any other person could comprehend the haunting thoughts, the feelings of isolation and the fear from loved ones. The women, throughout their pregnancy and, for many, after the delivery of their babies, experienced blame, fear, the cruelties of stigma, stereotyping and judging, and ultimately avoided any closeness or romantic relationships.

Many expressed drastic changes in their socialising patterns and that they spent more time alone. They did not socialise with friends in the way that they used to before they tested HIV positive. Others were able to cope as they relied on their spiritual beliefs.

Many blamed themselves for the trust that they had put in their partners. They felt that this trust had allowed them to forego an HIV test prior to initiating a sexual relationship with their current or ex-partners. Along with the blame, participants felt regret about their current pregnancy.

I’m not happy at all. To be honest, being pregnant now. I think it is a mistake. (P6)

Throughout pregnancy and breastfeeding, many participants were haunted by the fear of dying. Others lived with the fear of becoming ill and helpless and being hospitalised. They feared that the disease would progress and eventually lead to people knowing that they were HIV positive without them voluntarily disclosing their status.

The participants described the worst form of cruelty being a negative reaction from their partners after disclosing their HIV status. Some participants experienced rejection from their partners due to their disclosure, especially in cases where the partner tested HIV negative, while for others there was no obvious rejection but disclosure of the diagnosis nevertheless had negative relationship consequences.

After that when I got that results, I told him that I am positive. So he treated me some kind of treatment that were different, like he didn't eat in the house, didn't want to talk to me. Then I told him that I’m coming to the clinic and he didn't talk to me. When its end of the month he doesn't give me the money. So like I told myself that it's because I went to get tested and I’m positive. That is why he treats me different. (P9)

Partners proved unfaithful in some cases, which the participants were aware of. They feared that their partners would infect the women with whom they were having sexual relationships, but fearing judgement, they pondered the fact in silence.

He was seeing someone else, so I thought now it's the end of part one. Cause I’ve got two things to look at. I’m HIV positive and I’m worried about his girlfriend. (P3)

Some women were concerned about the health of their partners because they refused HIV testing or treatment.

But then I learnt that I have to be positive for him also because he was in denial … I’m scared to start the treatment whilst you are not on treatment because it obvious we are both infected. (P1)

Women felt isolated and wanted to keep their HIV diagnosis to themselves for fear of societal reactions towards them and their children. The company of their children was preferred to optimise the time that they had left while social gatherings were avoided out of fear that they would unintentionally disclose their HIV status and be rejected by their peers. Many found solace and acceptance in the privacy of their own homes and avoided society due to the cruelties of stigma.

#### The support of a few

All the women except one had disclosed their HIV status to their partners soon after the diagnosis. Some women had also disclosed their status to family members, colleagues or friends.

Even my friends that I was with was supporting me a lot. Even though I didn't talk to my family but my friends was supporting me a lot. (P6)

Support was found unconditionally for some and without difficulty, while others struggled with very little support. Participants perceived support from their mothers as positive and encouraging, while conversely some reactions of significant people in their lives were often painful and unexpected and made them feel that being HIV positive was their fault. The reaction of family members often left participants distressed and created the impression that the HIV diagnosis should not be discussed. Participants described situations where their partners distrusted them, sustaining the myth that HIV-positive women are promiscuous. Continued disbelief, lack of insight and negative reactions by partners led to participants reliving the diagnosis and the trauma thereof.

Then he says to me … ‘and your results? You have Aids you.’ He’ll say to me. He keeps throwing it in my face. (P8)

I told him. He didn't care much. He just took it as a sickness that somebody can go and apply for a grant and money. In the meantime I was sick. I saw it as a sickness. (P3)

Participants’ experience of health facilities differed. Some felt that health facilities inevitably disclosed their HIV status to the public because of queues and designated areas for receiving ART. This resulted in not attending the clinic closest to their home. Participants described healthcare workers as not understanding the pressures of participants’ work and their lives outside the clinic.

They don't understand that kuthi [us] we work for difficult people. (P7)

Others felt that the constant rotation of clinic staff through different areas of clinical care placed them at a disadvantage as they would develop a relationship with one healthcare worker and then would have to start anew at a subsequent visit when healthcare workers had changed. They also described a lack of trust in the ability of healthcare workers to maintain confidentiality. However, participants also described positive relationships with healthcare workers. Some valued the reassurance given by healthcare workers. They described talking to healthcare workers as a way of relieving their burden.

And then I think it's much nicer by the clinic because I know I’m going to reveal myself and I’m going to tell everything. And then that helps, so much because I know that I’ve off loaded my stuff. (P5)

Some participants felt that the lay counsellors needed more training on counselling skills as they were not happy with the counselling that they received. Participants also described receiving greater comfort from the professional nurses in the antenatal facility than the HIV lay counsellors. One participant also described a desire for nurses to do community outreaches to homes in order to address HIV-related stigma and encourage adherence.

I think that the clinic the thing that they can do, for those people who are affected, mmm, I think to visit them. I think it can help them a lot. Not to be scared to face anything. (P6)

#### A hope for a fractured future

Although participants experienced various challenges and uncertainties, they wanted to give birth to healthy children and live to see their future. For some, the initiation of ART gave them hope as they were confident that the treatment would improve their health, extend their life, protect their unborn children and even cure HIV.

Firstly I have to accept and knowing that yes HIV positive is not the end of the world. The thing that's gonna help me. I have to take the medicine in the right way. (P4)

I was happy because I told myself. I’m going to, this thing is going to be cured in my body. (P2)

However, for others the initiation of ART was a sign that they were very ill or a constant reminder that they were HIV positive. Many experienced side effects from the medication although these were mild and did not cause major discomfort. Taking ART also influenced decisions regarding employment.

I will never be able to work at Spar. Even if I wanted to. I can't work there. What is going to be my excuse every morning I start at seven? Before I go start work I must go and take my medication. (P8)

One participant who received ART from private suppliers (using her medical aid) found it more convenient and less disruptive to her lifestyle.

You got from day 1 to day 14 to collect your medication. So it doesn't disturb me in any way. I can even go after work. Go to work as usual then after work go and collect the medication. (P1)

Comparing HIV to other illnesses made it easier for some to hope for a future.

Like it's nothing, mmm, its nothing. It's the same like a headache … I think now I can control it. To be positive I take it as someone who has diabetes. Yes I’m taking it just like that. (P2)

The most important hope that they had for the future was that their unborn child would be HIV-free. They were concerned for the safety of the unborn child and some prayed to God that their child would be safe from HIV. Although many women had thought about having an abortion after the diagnosis, their feelings towards the pregnancy had changed as they experienced the physiological changes of pregnancy and then inevitably yearned for a healthy baby.

I wasn't happy at first, but now as she starting kicking I feel its relieving my stress. I’m feeling better. I’m starting to accept that he's there. Yes. (P6)

The safety of the child motivated them to take treatment and adhere to PMTCT programmes.

I didn't have a problem taking the treatment, because I knew I’m even protecting the child. (P3)

Clinic visits became a beacon of hope, and they described clinic visits as a way of improving their health and life.

When it's a day it's my date to come to the clinic, I wake up early in the morning about four o’clock. I’m awake. I’m hurry to get my treatment so that I can drink it. (P2)

## Discussion

Although the antenatal HIV prevalence in South Africa is plateauing, the prevalence in the Eastern Cape increased from 2011 to 2013 (National Department of Health, 2013). As seen in the findings of this study, many women only confronted the reality of HIV when diagnosed. They perceived lifestyle activities, such as having one sexual partner, as a protective factor against becoming HIV positive. In contrast, Kasenga et al. (2010) reported that women perceived themselves to be at a high risk of contracting HIV because their partners had sexual relationships with multiple women. This highlights the need for community-based education, motivation and skills programmes amongst women of child-bearing age that focus on empowering women to have healthy relationships. It further emphasises the need for active male partner involvement through psychoeducation (Weiss et al., 2014).

Being diagnosed with HIV during pregnancy brought with it all the challenges reported in the literature, such as negative emotions (for example, shock, disbelief and fear), a negative self-image, difficult decisions about disclosure, relationship difficulties, dealing with HIV-related stigma and becoming socially isolated (Gourlay et al., 2013). In addition, an HIV diagnosis affected women's feelings towards their unborn child. These feelings ranged from wanting an abortion and self-blame for putting the child at risk to a strong need to protect the unborn child. Acceptance of the diagnosis in order to reduce stress and avoid pregnancy complications was one way in which they dealt with this need, and many women also found support and encouragement in their religious beliefs. However, it was evident that some women were still reluctant to believe that they were HIV positive. Similarly, Katirayi et al. (2016) found that women in a Malawian study found it difficult to accept Option B+ without a CD4 count and some wanted a second HIV test.

In spite of these challenges, women diagnosed with HIV in pregnancy in a rural area seemed to have hope for the future. Several other studies identified this hope, which is grounded in the belief that ART will prolong pregnant women's lives and protect their unborn children through adherence to PMTCT programmes (Black et al., 2014; Elwell, 2016; Katirayi et al., 2016; Kim et al., 2016; Sanders, 2008). Some of the participants in this study felt that HIV was similar to other chronic diseases and that they could therefore control it if they took treatment. This may form the foundation of building resilience among HIV-positive pregnant and postpartum women. Even though women expressed this hope, however, there was continued uncertainty about the future that ongoing counselling should address.

There was a generally positive attitude towards taking ART. Although it was a reminder of being HIV positive and for some an indication that they were very ill, participants felt that it would help to improve their health. In Malawi, women reported similar experiences (Katirayi et al., 2016), but in rural Eastern Uganda, Rujumba et al. (2012) found that women equated an HIV diagnosis with death, regardless of the initiation of ART.

Participants in the current study did not report ART initiation on the same day as diagnosis as problematic and none mentioned that they had initially refused ART or stopped ART, whereas women in Malawi preferred obtaining permission from their husbands before starting ART or needed time to accept their HIV status (Katirayi et al., 2016; Kim et al., 2016). Partner support did not influence participants’ decisions about taking and continuing ART, unlike what was found by Kim et al. (2016) in Malawi.

Participants were, however, concerned about the stigma associated with taking ART and what it meant to be on ART. Their narratives clearly showed that these women might not have been in a fit psychological state to fully comprehend the implications of lifelong ART on the same day that they were diagnosed with HIV. Black et al. (2014) also found that women who had to rapidly initiate ART late in pregnancy accepted ART initiation but that it was difficult to manage the dual challenges of being diagnosed with HIV and being dependent on lifelong ART at the same time. Medication side effects did not significantly disturb participants in our study whereas it was a significant contributor to women stopping ART in Malawi (Kim et al., 2016).

Disclosure is one of the key factors influencing adherence. In this study, all women except one had disclosed their status to their partners soon after the HIV diagnosis. This is contrary to findings reported by Rujumba et al. (2012) that disclosing an HIV diagnosis to a partner was too difficult and that women avoided disclosure because they did not want to cause stress for their partners. A study in Mpumalanga, South Africa, found that just under 50% of women disclosed their serostatus to partners throughout the course of a couples intervention and further stated that this may be due to gender inequalities (Weiss et al., 2014). It is not clear why the majority of participants in this study had disclosed their status to partners. After disclosure, there was an expectation by the women in the current study that their partners would undergo an HIV test as well. For many, disclosure led to being blamed for being HIV positive, conflict in the relationship and a lack of support, which was also reported in Malawi by Elwell (2016).

As soon as the partner had been diagnosed HIV positive, concern was transferred to the well-being of the partner instead of the well-being of the participant. Two participants experienced emotional abuse from their partners, but none of the participants experienced physical abuse, as found by Kalembo et al. (2013). Kim et al. (2016) found that husbands sometimes refused to allow women to go to the clinic or threw away their tablets. There is a growing body of evidence indicating that HIV-positive women are more likely to experience intimate partner violence and are consequently more likely to be less adherent to treatment and to have poorer health outcomes (Nava, 2012).

Some participants were dissatisfied with the quality of the counselling that they received from lay healthcare workers in the facility. In Malawi, healthcare workers and pregnant women expressed similar concerns, and a lack of thorough counselling to inform women's decisions and poor interactions with healthcare workers were identified (Elwell, 2016; Katirayi et al., 2016).

Having to attend an antenatal clinic was not changed by having an HIV diagnosis in that the women still had to attend routine antenatal and postnatal visits. However, the structure and outlay of the health facility concerned participants as they feared inevitable disclosure of their HIV status if they collect treatment at the part of the facility dedicated for HIV care. This was also identified as problematic to women in Malawi (Elwell, 2016). Two participants in this study stated that they had no confidence in the ability of healthcare workers to maintain confidentiality, although none reported an incident in which confidentiality had been breached by healthcare workers – as was described in a study in Northern Ireland (Kelly, Alderdice, Lohan, & Spence, 2013). Structural barriers and poor relationships with healthcare workers have also been reported in other studies (Elwell, 2016; Gourlay et al., 2013; Katirayi et al., 2016).

Issues specific to rural areas previously reported in the literature such as access to services and culture-specific issues such as having to ask permission from a husband before starting ART (Gourlay et al., 2013; Katirayi et al., 2016) were not reported by women in this study. Although women reported that it was sometimes inconvenient to attend the clinic due to work or that taking ART affected employment decisions, none of them reported transport or access problems. On the contrary, they preferred to attend clinics farther away from their homes to avoid HIV-related stigma. One participant reported using a private healthcare provider, which made accessing treatment more convenient. Although this may not be feasible for all women, since they do not have medical aid, it may be worthwhile for the South African public sector to engage private partners to make ART more accessible to pregnant women.

These findings clearly indicate that the decisions by HIV-positive pregnant women regarding participation in PMTCT programmes are influenced by their unique sociocultural context. This necessitates contextually focused counselling interventions.

## Limitations

The study was performed at one clinic in the Alfred Ndzo health district of the Eastern Cape province. Clients from other clinics may have had different experiences. Due to the sensitivity of an HIV diagnosis and the need to protect their current pregnancies and unborn children from any undue stress, some participants may have withheld experiences. Additionally, only women who attended antenatal and postnatal visits were recruited and women who did not return for routine antenatal and postnatal clinic visits after being diagnosed were excluded.

## Conclusions

Ultimately, women diagnosed with HIV during pregnancy were concerned about the well-being of their unborn children, and this concern motivated their adherence to ART. This finding resonates with the findings from recently published studies conducted elsewhere in sub-Saharan Africa. Women's lived experiences are situated in their unique sociocultural context, and although some known challenges remain, counselling and support strategies need to be informed by exploring context-specific issues and involving the local community. The high burden of HIV and the increased number of women being tested for HIV during pregnancy may cause healthcare workers to become ‘indifferent’ to the individual experiences and circumstances of women. It is important to remember that many of the known and well-described issues related to an HIV diagnosis continue to affect the lives of women and cannot be overlooked.

Recommendations for practice include skills development of healthcare workers on testing and counselling procedures; implementation of a follow-up counselling schedule, including partner involvement; integration of HIV and antenatal/postnatal care services to avoid stigma and promote continuity of services; and peer-led community support groups. These are similar to recommendations made in other recently published work (Elwell, 2016; Katirayi et al., 2016).
